# Approach to fever in children among final-year nursing students: a multicenter survey

**DOI:** 10.1186/s12912-023-01263-3

**Published:** 2023-04-13

**Authors:** Gregorio P. Milani, Antonio Corsello, Marta Fadda, Ilaria Falvo, Mario G. Bianchetti, Diego Peroni, Elena Chiappini, Barbara Cantoni, Patrizio Sannino, Anne Destrebecq, Paola Marchisio

**Affiliations:** 1 Department of Clinical Sciences and Community Health, Università degli Studi di Milano, Milan, Italy; 2 Pediatric Unit, Fondazione IRCCS Ca’ Granda Ospedale Maggiore Policlinico, via della Commenda 9, Milan, 20122 Italy; 3 Institute of Public Health, Faculty of Biomedical Sciences, Università della Svizzera italiana, Lugano, Switzerland; 4 Faculty of Biomedical Sciences, Università della Svizzera Italiana, Lugano, Switzerland; 5 Department of Clinical and Experimental Medicine, Section of Pediatrics, University of Pisa, Pisa, Italy; 6 Pediatric Infectious Disease Unit, Department of Health Sciences, Meyer Children’s University Hospital, University of Florence, Florence, Italy; 7 Direzione Professioni Sanitarie, Fondazione IRCCS Ca’ Granda Ospedale Maggiore Policlinico, Milan, Italy; 8 Department of Biomedical Sciences for Health, University of Milan, Milan, Italy; 9 Department of Pathophysiology and Transplantation, University of Milan, Milan, Italy

**Keywords:** Education, Children, Fever phobia, Inappropriate treatment, Evidence, Physical methods, Education

## Abstract

**Background:**

Unfounded concerns regarding fever are increasingly observed among nurses worldwide. However, no study has so far explored the preferred approach towards pediatric fever among nursing students. Therefore, we aimed to investigate the attitude towards pediatric fever among final-year nursing students.

**Methods:**

Between February and June 2022, final-year nursing students of 5 Italian university hospitals were asked to answer an online survey on their approach to fever in children. Both quantitative and qualitative methods were utilized. Multiple regression models were employed to explore the existence of moderators on fever conceptions.

**Results:**

The survey was filled in by 121 nursing students (response rate 50%). Although most students (98%) do not consider discomfort to treat fever in children, only a minority would administer a second dose of the same antipyretic in nonresponsive cases (5.8%) or would alternate antipyretic drugs (13%). Most students would use physical methods to decrease fever (84%) and do not think that fever has mainly beneficial effects in children (72%). The own know-how adequacy on fever was inversely associated (OR 0.33, 95% CI 0.13–0.81) with the beliefs that high fever might lead to brain damage. No further predictive variable was significantly associated with the concern that fever might be associated with brain damage, the advice of physical methods use, and the assumption that fever has mostly positive effects.

**Discussion:**

This study shows for the first time that misconceptions and inappropriate attitudes towards fever in children are common among final-year nursing students. Nursing students could potentially be ideal candidates for improving fever management within clinical practice and amongst caregivers.

**Supplementary Information:**

The online version contains supplementary material available at 10.1186/s12912-023-01263-3.

## Background

Fever in children is one of the most frequent complaints for care seeking [1]. It usually results from an infection and is a cardinal part of the inflammatory response [2]. As such, fever has a relevant role in fighting infection [3]. However, since defense mechanisms can go awry in a few cases, fever can be associated with an increased metabolic rate and distress, and affect the overall wellbeing of a child [4]. Therefore, the primary goal of fever treatment is to improve the child’s comfort rather than modifying the body temperature [5].

Unfounded concerns and inappropriate attitudes towards fever have been increasingly observed among nurses [6], who play a key role both in the care of feverish children and in providing the information to caregivers [7]. For instance, several studies conducted among nurses have reported the use of physical antipyresis (such as sponges using cold water) or alternating antipyretic drugs to reduce body temperature [8–10]. These non-evidence-based behaviors, which mainly rely on the assumptions that fever is per se noxious, fail in the pursuit of decreasing body temperature and put children at risk of side effects (e.g., distress or drug overdose) [11, 12].

Despite educational interventions implemented to address this issue [10], a gap still exists between the available recommendations and attitudes towards fever among nurses [13, 14]. Nursing students might represent an ideal population for interventions aimed at transferring evidence-based recommendations into day-to-day clinical practice [13, 14].

However, no study has so far explored the preferred approach towards pediatric fever among nursing students. For this purpose, we carried out a survey to investigate the attitude towards pediatric fever among final-year nursing students.

## Methods

Between February and June 2022, we conducted a cross-sectional study among final-year nursing students of 5 Italian university hospitals: Fondazione IRCCS Ca’ Granda Ospedale Maggiore Policlinico (Milan), Presidio Ospedaliero, ASST Rhodense (Rho), Istituto Nazionale dei Tumori (Milan), Ospedale Maggiore di Crema (Crema), and ASST Valle Olona (Busto Arsizio). Students were invited to answer a survey composed by both closed and open-ended questions regarding fever in otherwise healthy children.

A link to access an anonymous questionnaire on Google Form was sent to the institutional email of all eligible participants. Two further reminders were sent after 10 and 20 days, respectively.

To develop the questionnaire, we initially reviewed the seminal survey on child fever proposed by Barton D. Schmitt [15]. This survey has been utilized in over 70 studies globally [6]. Next, the survey was modified slightly by four international experts on child fever (M.F., M.G.B., E.C., and P.M.) and adapted for nursing students. The modified survey was then tested by six researchers, including two nurses, two board-certified pediatricians, and two science communicators, and two questions were slightly revised based on their feedback. Finally, two students completed the developed questionnaire independently on two occasions, with a 10-day interval between each administration. The intra-rater reproducibility was found to be 98%.

Briefly, the following information was collected by 15 close-ended questions: (1) age and gender; (2) training related to fever received during the nursing program and own know-how adequacy on fever; (3) attitude towards fever management in children. Six questions included Likert scale answers (“Very inadequate”, “Inadequate”, “Adequate” and “Very adequate”, or “Very unlikely”, “Unlikely”, “Likely” and “Very likely”, or “Strongly disagree”, “Disagree”, “Agree”, “Strongly agree”). Moreover, open-ended questions explored second-hand or personal relevant experience related to child’s fever. The full questionnaire is provided in the online supplementary material.

### Statistical analysis

Data are reported as absolute frequency and percentage or as median and interquartile, as appropriate. Dichotomous variables were compared by Fisher exact test.

To investigate the existence of moderators on fever conceptions, the 4-digit scales of the answers related to (1) possible brain damage associated to fever, (2) the use of physical methods to decrease body temperature and (3) the beneficial effects of fever, were collapsed into 2-digit measures. Multiple logistic regression models were employed to assess the associations between the answers to the above-mentioned issues (dependent variables) and age, gender, teaching time on fever (< 1 vs. ≥ 1 h), own know-how adequacy on fever (“very inadequate” + “inadequate” vs. “adequate” + “very adequate”), second-hand or personal relevant experience related to child’s fever (independent variables). Odds ratios (ORs) with 95% confidence intervals (95% CIs) were calculated. A two-tailed p < 0.05 was assumed as significant. Statistical analyses were performed using “R” program (version 3.5.3, 2019).

### Qualitative analysis

An inductive-deductive analysis of all open-ended answers was performed to define experiential patterns by two authors (MF and IF). The analysis comprised four phases: (1) data cleaning and data reorganization, during which we differentiated between personal and second-hand experiences; (2) inductive analysis, during which we developed categories that represented frequent content patterns; (3) deductive analysis, where we reduced and re-labelled the categories to avoid redundancies and deductively coded all answers accordingly; and (4) thematic analysis in which we identified overarching themes and organized the results under four main types of experience. This approach is consistent with the thematic approach described by Braun and Clarke [16, 17]. Further details are described elsewhere [18].

### Ethical statement

The study was approved by the Institutional Ethics Committee of the University of Milan. Since no patient was involved, a single Ethics Committee approval was considered sufficient. All enrolled students provided a written informed consent to participate. No incentive or compensation was given to participants who full-filled the survey. The study was conducted according to the principles of Helsinki declaration and later amendments.

## Results

A total of 241 final-year nursing students were invited to participate and 121 filled-in the survey (response rate 50%). Most participants were female (N = 100, 82%). The median age of participants was 24 [interquartile range: 22–25] years. Many students evaluate their own know-how on fever (Table 1) as adequate o very adequate (N = 69, 57%). Although most nursing students (N = 115, 98%) do not consider discomfort rather than body temperature to treat fever in children (Table 2), only a minority would administer a second dose of the same antipyretic in nonresponsive cases (N = 7, 5.8%) or would altern antipyretics (N = 16, 13%). Most students do not think that fever can lead to brain damage (N = 89, 74%). On the other hand, most participants would use physical methods to decrease fever (N = 101, 83%) and do not think that fever has mainly beneficial effects in children (N = 87, 72%), as shown in Table 3.

**Table 1:** Characteristics of the 121 nursing students participating the study. Data are given as median and interquartile range or absolute frequency and percentage

Age, years	24 [22–25]
Gender	
Males	21 (17)
Females	100 (83)
Teaching time dedicated to fever	
>5 h	4 (3.3)
>2–5 h	22 (18)
1–2 h	46 (38)
<1 h	31 (26)
No time	18 (15)
Topic fever deepened by means of a textbook, yes	68 (56)
Own know-how adequacy on fever	
Very adequate	11 (9.0)
Adequate	58 (48)
Inadequate	48 (40)
Very inadequate	4 (3.3)

**Table 2:** Management of fever in children by nursing students. Data are given as absolute frequency and percentage

N	121
Criterion for treating fever in children	
Body temperature	
>36.7 °C	0 (0.0)
>37.4 °C	26 (14)
>37.9 °C	59 (47)
>38.4 °C	28 (32)
>38.9 °C	3 (4.9)
I would consider discomfort rather than the temperature	6 (2.4)
Most prescribed antipyretic drug	
Acetaminophen	110 (91)
Ibuprofen	5 (4.1)
Salicylates	6 (5.0)
Second dose of the same antipyretic in nonresponsive cases	
Very likely	7 (5.8)
Likely	0 (0.0)
Unlikely	64 (53)
Very unlikely	50 (41)
Alternating antipyretic regimen	
Very likely	1 (0.8)
Likely	15 (12)
Unlikely	53 (44)
Very unlikely	52 (43)

**Table 3:** Answers of nursing students to questions on the assumption that fever can cause cerebral damage, on the use of physical methods to decrease the fever and on the belief that fever has mostly positive effects in children. Data are given as absolute frequency and percentage

N	121
Fever can cause cerebral damage	
Very likely	6 (5.0)
Likely	26 (21)
Unlikely	80 (66)
Very unlikely	9 (7.4)
Use of physical methods	
Very likely	41 (34)
Likely	60 (50)
Unlikely	16 (13)
Very unlikely	4 (3.3)
Fever has mostly beneficial effects	
Strongly agree	1 (0.8)
Agree	33 (27)
Disagree	70 (58)
Strongly disagree	17 (14)

Nine and 15 respondents reported a relevant personal episode of fever from their childhood or about another child, respectively. The open answers pointed out that nursing students had relevant experiences associated to high body temperature, complications of fever (e.g., febrile seizures), etiology of fever, and management of high body temperature (details are provided in the supplementary online material). High body temperature and consequences of fever are the most frequently relevant experiences disclosed by participants.

The full results of the qualitative analyses (eTable 1) and of the logistic regression models (eTable 2–4) are reported within the online supplementary material. An adequate/very adequate own know-how on fever was inversely associated (OR 0.33, 95% CI 0.13–0.81) with the beliefs that high fever might lead to brain damage. No further predictive variable was significantly associated with the concern that high fever might lead to brain damage, the advice of physical methods and the assumption that fever has mostly positive effects.

## Discussion

This is the first study investigating the approach to pediatric fever among nursing students. The results indicate that misconceptions and improper approach towards fever in children are prevalent among final-year nursing students.

More than 40 years ago, Schmitt administered a questionnaire to caregivers of children visiting a hospital in Denver and noted unrealistic concerns about the possible consequences of fever. This attitude was also associated to a tendency to overtreat fever in most of the respondents [15]. Subsequent studies showed that this phenomenon, also known as “fever phobia”, was not limited to caregivers, but was common even among nurses in many countries [19–22]. This study reveals that merely 5% of nursing students view fever as a beneficial mechanism for children, with the majority instead recommending non-evidence-based interventions to reduce body temperature. These findings highlight the presence of fever phobia also within the nursing student population.

**Fig. 1:**
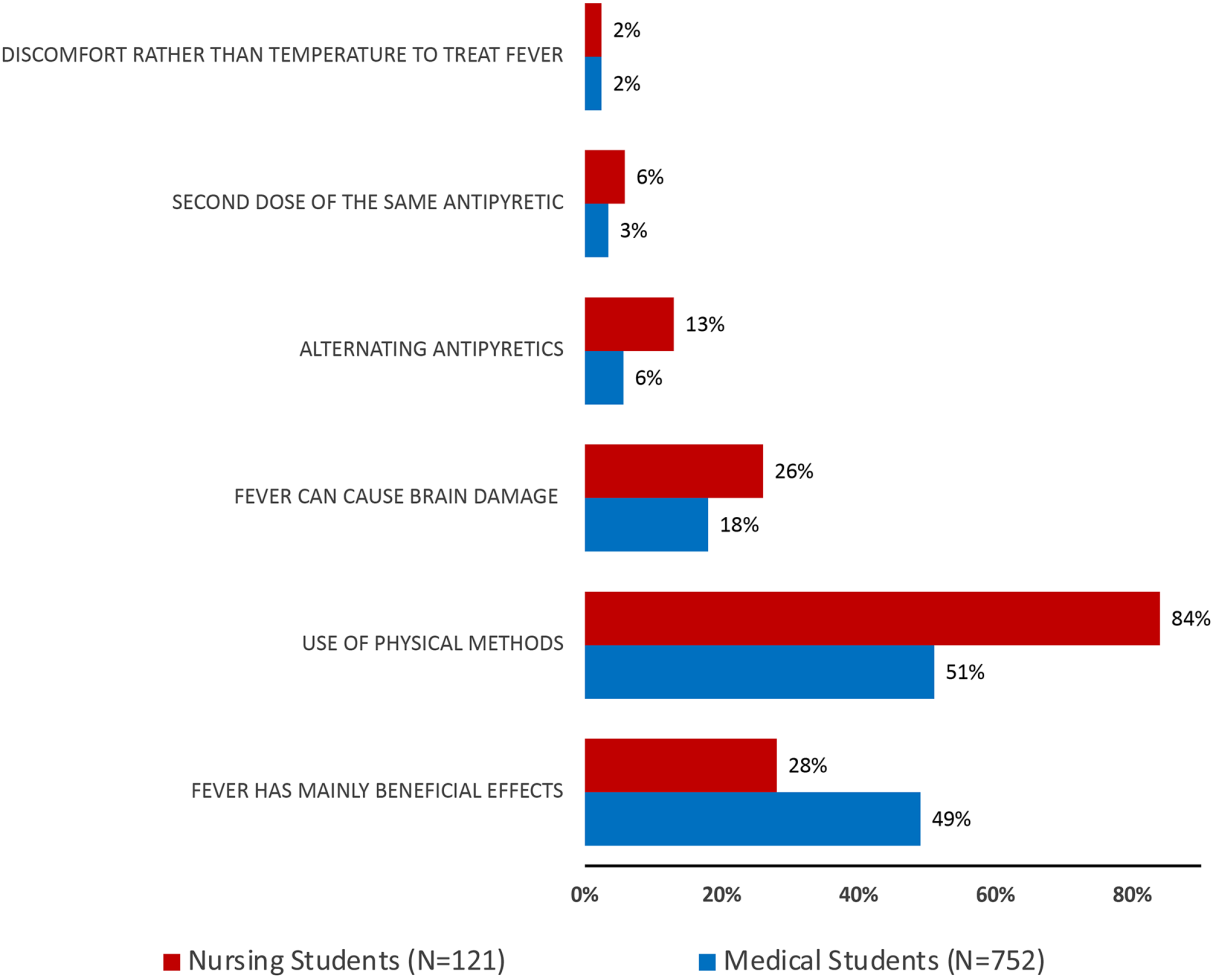
Conceptions and attitudes of nursing and medical students [18]

Several results from this survey are consistent with those of a previous study [18] we conducted among final-year medical students. Like medical students, a minority of nursing students consider discomfort, rather than body temperature, to be the primary indicator for treating fever (Fig. 1). However, more than 80% of both nursing and medical students would not administer an additional dose of antipyretic medication or switch to another antipyretic drug for a child with a persistently high body temperature. Furthermore, approximately 20% of nursing and medical students believe that fever can lead to brain damage, and more than half recommend physical methods to reduce body temperature. Finally, the lack of an association between misconceptions and personal or second-hand experiences related to childhood fever in both groups of students supports the notion that individual learned traits have a negligible impact on the approach to fever [23]. Conversely, this study indicates that a minority of nursing students believe that fever is beneficial to children, whereas approximately half of the medical students (49%) agree with this evidence. These findings indirectly support the results of a recent systematic review, which suggests that misconceptions about fever are similarly prevalent among physicians and nurses, with the exception that nurses are less aware of the potential beneficial effects of fever [6].

The results of this study emphasize the potential benefits of training in the management of fever. A majority of nursing students (> 70%) reported a limited amount of teaching time (less than 1 h or no time at all) devoted to child fever during their academic training. On the other hand, the only variable found to be associated with less frequent beliefs that fever may result in brain damage was the perception of an adequate knowledge on this subject. These data are relevant, given the crucial role that nurses play in educating parents about fever and managing it in various healthcare contexts, including outpatient clinics, emergency units, and pediatric wards [7]. If subsequent international surveys confirm the results of this study, then training on the management of child fever should receive greater attention in nursing academic programs worldwide.

This study has limitations. It was conducted exclusively in Italy and the sample size was limited. Furthermore, the responses provided by the students might not entirely represent the day-to-day clinical approach and might have been influenced by the desire to respond in a socially acceptable way. Finally, we could not investigate the possible role of regional differences on fever approach.

## Conclusion

This study shows that misconceptions and inappropriate attitudes towards fever in children are common among final-year nursing students. This population might become an ideal candidate to improve fever management in the clinical practice and among caregivers, as the conceptions and behaviors of students play a crucial role in shaping their future work [24]. To this end, future research should address the impact of educational interventions on nursing practice going forward.

### Electronic supplementary material

Below is the link to the electronic supplementary material.

Supplementary Material 1

## Data Availability

upon reasonable request to the corresponding author.
