# Supplementary material for: Approach to fever in children among final-year nursing students: a multicenter survey
Source: BMC Nurs. 2023 Apr 13;22:119. doi: 10.1186/s12912-023-01263-3 (PMC10100172; doi:10.1186/s12912-023-01263-3)
Supplement: Supplementary file 1 — Supplementary Material 1 [file 12912_2023_1263_MOESM1_ESM.docx]

**Supplementary online Material**

**Supplementary file 1**

**Questionnaires**

The original questionnaire was in Italian

1) Gender

2) How old are you?

3) Which hospital are you attending?

4) How many hours of lessons were focused on fever during the medical course?

- >5 hours
- >2-5 hours
- 1-2 hours
- < 1 hour
- No time

5) Did you read any section in a textbook dedicated to fever?

- Yes
- No

6) How adequate do you think that your knowledge on fever is adequate for your future medical work?

- Very adequate
- Adequate
- Inadequate
- Very inadequate

7) Which criteria would you employ for treating the fever in children?

- Body temperature >36.7°C
- Body temperature >37.4°C
- Body temperature >37.9°C
- Body temperature >38.4°C
- Body temperature >38.9 °C
- I would consider discomfort rather than the temperature

8) Which of these drugs would you prescribe as first choice to treat fever in children?

- Acetaminophen
- Ibuprofen
- Salicylates

9) How likely would you be to administer a further antipyretic dose if the fever does not decrease quickly after a dose of an antipyretic (e.g., within 30 minutes)?

- Very likely
- Likely
- Unlikely
- Very unlikely

10) How likely would you be to administer a different antipyretic drug if the fever does not decrease quickly after the administration of an antipyretic (e.g., within 30 minutes)?

- Very likely
- Likely
- Unlikely
- Very unlikely

11) How likely do you think that high fever might lead to brain damage in children?

- Very likely
- Likely
- Unlikely
- Very unlikely

12) How likely would you advise physical methods (e.g. bathing or sponging) for treating child’s fever?

- Very likely
- Likely
- Unlikely
- Very unlikely

13) To what extent do you agree with the statement that “fever has mostly beneficial effects on children”?

- Strongly Agree
- Agree
- Disagree
- Strongly disagree

14) Do you remember or did anyone tell you about an important episode of fever when you were a child?

- Yes
- No

If yes, can you tell us more about this experience?

15) Do you remember or did anyone tell you about an important episode of fever of any other child?

- Yes
- No

If yes, can you tell us more about this experience?

**Qualitative analysis -methods**

The analysis of the open answers was conducted in four main phases:

First phase - data cleaning and data reorganization

Data was reorganized within an Excel document into three different sheets: sample, personal experiences and second-hand experiences. Six responses were excluded as they did not contain any text and one response to the personal experience question was treated as a second-hand experience answer.

Second phase - inductive approach

IF read the open answers multiple times and started to develop categories that represented frequent content patterns. The analysis allowed the identification of 14 categories for personal experiences and 15 categories for second-hand experiences.

Third phase - deductive approach

Categories were reduced and re-labelled following agreement between IF, GPM and MF to avoid redundancies. This was done during multiple meetings. All answers were deductively coded following this categorization.

Fourth phase - thematic analysis

The frequency of the categories and the associated percentage were calculated. All team members met to identify overarching themes, resulting in the organization of the results under four main types of experience.

**Qualitative results - results**

**eTable 1:** Personal and second-hand experiences with fever

Personal experiences with fever (N=9)

| **Types of experience** | **Description of the experience** | **Examples** | **N (%)** |
| --- | --- | --- | --- |
| Clinical complication(s) of fever | Neurological symptoms | Seizures | 2 (22) |
|  | Hospital contact | Access to the emergency room or medical guard service | 1 (11) |
| Etiology of fever | Viral or bacterial infection | Meningitis  Pneumonia | 1 (11)  1 (11) |
| Body temperature | High temperature | High temperature | 4 (44) |

Second-hand experience with fever (N=15)

| **Types of experience** | **Description of the experience** | **Examples** | **N (%)** |
| --- | --- | --- | --- |
| Clinical complication(s) of fever | Neurological symptoms | Seizures, loss of consciousness, loss of motor and cognitive ability | 10 (67) |
|  | Psychiatric symptoms | Allucinations | 2 (13) |
| Treatment of fever | Fever’ management with non-scientific practices, dangerous management | Use of sponge water | 1 (6.7) |
|  | Viral or bacterial infection | Pericarditis | 1 (6.7) |
| Body temperature | High temperature | High temperature | 1 (6.7) |

**Results of the logistic regression models**

**eTable 2.** Logistic regression model on the beliefs that high fever might lead to brain damage (dependent variable). Age, gender, own know-how adequacy on fever, teaching time on fever, history of relevant personal or second-hand experience with child’s fever were the predictive variables. Data are reported as Odds ratio (OR) and 95% Confidence Interval (CI).

|  | **OR** | **95% CI** | **P** |
| --- | --- | --- | --- |
| Age (years) | 0.96 | 0.86-1.06 | 0.42 |
| Gender (male) | 2.70 | 0.92-7.95 | 0.07 |
| Own know-how adequacy on fever  (very adequate + adequate) | 0.33 | 0.13-0.81 | 0.01 |
| Teaching time (<1 hour) | 1.05 | 0.42-2.62 | 0.92 |
| Personal experience (yes) | 0.88 | 0.18-4.41 | 0.88 |
| Second hand experience (yes) | 1.41 | 0.38-5.22 | 0.61 |

**eTable 3.** Logistic regression model on the advice to use physical methods for treating child’s fever (dependent variable). Age, gender, own know-how adequacy on fever, teaching time on fever, history of relevant personal or second-hand experience with child’s fever were the predictive variables. Data are reported as Odds ratio (OR) and 95% Confidence Interval (CI).

|  | **OR** | **95% CI** | **P** |
| --- | --- | --- | --- |
| Age (years) | 0.93 | 0.89-1.11 | 0.90 |
| Gender (male) | 0.91 | 0.25-3.30 | 0.89 |
| Own know-how adequacy on fever  (very adequate + adequate) | 1.41 | 0.50-3.93 | 0.51 |
| Teaching time (<1 hour) | 2.00 | 0.70-5.70 | 0.20 |
| Personal experience (yes) | 2.18 | 0.24-20.10 | 0.49 |
| Second hand experience (yes) | 2.74 | 0.33-23.00 | 0.35 |

**eTable 4.** Logistic regression model on the assumption that fever has mainly positive effects in children (dependent variable). Age, gender, own know-how adequacy on fever, teaching time on fever, history of relevant personal or second-hand experience with child’s fever were the predictive variables. Data are reported as Odds ratio (OR) and 95% Confidence Interval (CI).

|  | **OR** | **95% CI** | **P** |
| --- | --- | --- | --- |
| Age (years) | 0.99 | 0.90-1.10 | 0.99 |
| Gender (male) | 1.18 | 0.39-3.61 | 0.77 |
| Own know-how adequacy on fever  (very adequate + adequate) | 1.55 | 0.65-3.73 | 0.32 |
| Teaching time (<1 hour) | 0.54 | 0.22-1.29 | 0.16 |
| Personal experience (yes) | 0.24 | 0.03-2.09 | 0.20 |
| Second hand experience (yes) | 0.93 | 0.26-3.30 | 0.91 |
